# Involvement of the P2X7-NLRP3 axis in leukemic cell proliferation and death

**DOI:** 10.1038/srep26280

**Published:** 2016-05-25

**Authors:** Erica Salaro, Alessia Rambaldi, Simonetta Falzoni, Francesca Saveria Amoroso, Alessia Franceschini, Alba Clara Sarti, Massimo Bonora, Francesco Cavazzini, Gian Matteo Rigolin, Maria Ciccone, Valentina Audrito, Silvia Deaglio, Pablo Pelegrin, Paolo Pinton, Antonio Cuneo, Francesco Di Virgilio

**Affiliations:** 1 Department of Morphology, Surgery and Experimental Medicine, Section of Pathology, Oncology and Experimental Biology, University of Ferrara, Italy; 2 Department of Medical Sciences, Section of Hematology, University of Ferrara, Italy; 3 Department of Medical Sciences, Immunogenetics Unit, Human Genetics Foundation, Torino, Italy; 4 Murcia Biomedical Research Institute (IMIB), Hospital Virgen de la Arrixaca, 30120 Murcia, Spain

## Abstract

Lymphocyte growth and differentiation are modulated by extracellular nucleotides and P2 receptors. We previously showed that the P2X7 receptor (P2X7R or P2RX7) is overexpressed in circulating lymphocytes from chronic lymphocytic leukemia (CLL) patients. In the present study we investigated the P2X7R/NLRP3 inflammasome axis in lymphocytes from a cohort of 23 CLL patients. P2X7R, ASC and NLRP3 were investigated by Western blot, PCR and transfection techniques. P2X7R was overexpressed and correlated with chromosome 12 trisomy in CLL patients. ASC mRNA and protein were also overexpressed. On the contrary, NLRP3 was dramatically down-modulated in CLL lymphocytes relative to lymphocytes from healthy donors. To further investigate the correlation between P2X7R, NLRP3 and cell growth, NLRP3 was silenced in THP-1 cells, a leukemic cell line that natively expresses both NLRP3 and P2X7R. NLRP3 silencing enhanced P2X7R expression and promoted growth. On the contrary, NLRP3 overexpression caused accelerated apoptosis. The P2X7R was also up-modulated in hematopoietic cells from NLRP3-KO mice. In conclusion, we show that NLRP3 down-modulation stimulates P2X7R expression and promotes growth, while NLRP3 overexpression inhibits cell proliferation and stimulates apoptosis. These findings suggest that NLRP3 is a negative regulator of growth and point to a role of the P2X7R/NLRP3 axis in CLL.

The NLRP3 inflammasome has become a focus of hot interest for its ability to modulate cell responses to a wide array of exogenous or endogenous injurious agents^1^. The most important plasma membrane receptor responsible for NLRP3 activation is P2X7R^2^. P2X7R drives NLRP3 recruitment at discrete intracellular sites and activation via an as yet poorly understood mechanism^3^. The most important pathophysiological response triggered by activation of the P2X7R/NLRP3 inflammasome axis is release of the pro-inflammatory cytokine IL-1β^4^. However, over the last few years it has been clearly shown that P2X7R can also support cell growth^5^,^6^,^7^,^8^. *In vitro* and *in vivo* observations show that P2X7R expression increases the endoplasmic reticulum Ca^2+^ content and the mitochondrial potential, enhances intracellular ATP levels, activates NFATc1, prevents apoptosis and promotes cell proliferation^5^,^9^.

We previously showed that P2X7R is upregulated in peripheral lymphocytes from patients affected by chronic lymphocytic leukemia (CLL)^10^. In addition, *in vivo* studies show that P2X7R overexpression accelerates, while on the contrary its down-modulation inhibits tumor growth^6^. NLRP3 is known to cause “pyroptosis”^11^, a type of caspase-1-dependent programmed necrosis often observed following infection by intracellular pathogens (e.g. Salmonella), or “pyronecrosis”^12^, a caspase-1-independent cell death. It has been proposed that NLRP3 might function as a negative regulator of tumorigenesis in colitis-associated cancer^13^,^14^, or as a contributing factor to melanoma spreading due to autoinflammation^15^, and a recent study shows that the NLRP3 protein is either down-regulated or completely lost in hepato-cellular carcinoma^16^. However, an in depth investigation of the contribution of NLRP3 to cancer cell growth has never been carried out^17^,^18^. While it is clear that NLRP3 might interfere with cancer cell growth by modulating the inflammatory state of the tumor microenvironment, it cannot be excluded that NLRP3 itself has a direct role, acting on intracellular pathways responsible for the modulation of cell growth or apoptosis.

In this study, we investigated P2X7R and NLRP3 expression and function in B lymphocytes from CLL patients and their role in cell growth. Our data show that P2X7R and ASC are overexpressed while NLRP3 is strongly down-modulated in CLL lymphocytes. P2X7R expression closely correlates with chromosome 12 trisomy, a known adverse prognostic factor in CLL. Furthermore, NLRP3 down-modulation drives P2X7R expression and promotes growth. On the contrary, NLRP3 overexpression stimulates apoptotic caspase activity and cell death. In conclusion, our findings identify NLRP3 as a novel growth-inhibiting factor of potential relevance in CLL.

## Results

### NLRP3 is down-modulated in CLL lymphocytes

**Figure 1:**
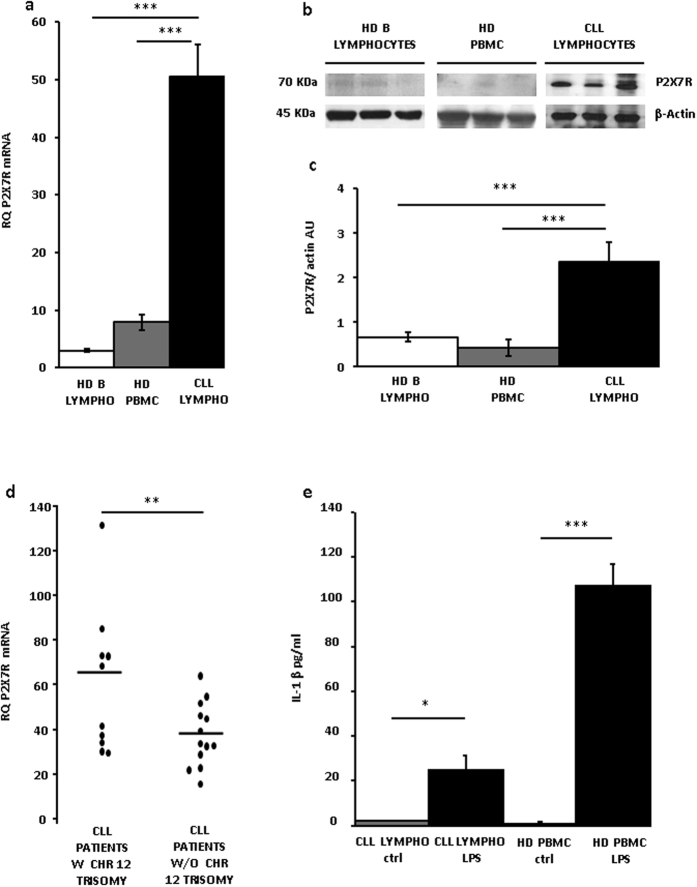
P2X7R expression in CLL lymphocytes. (**a**) P2X7R mRNA level was evaluated by Real-Time PCR as described in Methods. P2X7R expression was normalized on G3PDH internal control and displayed as fold increase. Data are shown as mean ± SEM, n = 3, ***p < 0.001. (**b**) Representative Western blot of P2X7R from three subjects representative of each population. (**c**) Densitometry of P2X7R normalized on actin level (AU, arbitrary Units). N = 3, ***p < 0.001. (**d**) P2X7R mRNA expression in lymphocytes from CLL patients with or without chromosome 12 trisomy; **p < 0.01. (**e**) IL-1β release (see Methods) was evaluated by ELISA from CLL lymphocytes and HD PBMCs. Cells were overnight (12 h) incubated in the presence of LPS. Data are averages ± SEM, *p < 0.05; ***p < 0.001.

We previously showed that peripheral blood monocytes (PBMCs) from CLL patients up-modulate P2X7R mRNA and protein^10^. This observation suggested that the P2X7R might be involved in leukemogenesis, and might even be a marker of the disease^7^. In full agreement with our previous findings^10^ Fig. 1a shows that leukemic lymphocytes expressed P2X7R mRNA to a level at least six fold higher than PBMCs from healthy donors (HD), and almost 15 fold higher than purified HD peripheral blood B lymphocytes. Over-expression of P2X7R mRNA was paralleled by overexpression of the P2X7R protein, as shown by an exemplificative Western blot, and corresponding densitometry, of CLL lymphocytes, HD PBMCs and peripheral blood B lymphocytes (Fig. 1b,c). Next we asked whether the increased P2X7R expression level might correlate with known CLL prognostic factors. The *P2RX7* gene is located on chromosome 12q24, that is duplicated in about 16% of CLL patients (chromosome 12 trisomy), and is a known adverse prognostic factor. Therefore, we analyzed whether P2X7R overexpression was associated with chromosome 12 trisomy. As shown in Fig. 1d, there was a statistically significant (**p < 0.01) higher P2X7R mRNA level in patients with versus patients without chromosome 12 trisomy, suggesting a gene-dosage effect.

The P2X7R is a main switch for processing and release of the potent pro-inflammatory cytokine IL-1β, thus we asked whether CLL lymphocytes, due to increased P2X7R expression might be endowed with IL-1β-releasing activity. Previous literature reports suggested that this might not be the case as IL-1β plasma levels are lower in CLL than in healthy controls^19^. Figure 1e, shows that IL-1β release from quiescent lymphocytes was negligible, whether from HD or CLL subjects. Notably, LPS-stimulated release was about four fold lower in CLL lymphocytes than in HD PBMCs despite much higher P2X7R expression in the former cells. The P2X7R is tightly functionally associated to the NLRP3 inflammasome, the major intracellular apparatus responsible for IL-β processing and release^2^, therefore, we investigated whether ASC and NLRP3 expression paralleled P2X7R expression level, and thus was also increased in CLL lymphocytes.

**Figure 2:**
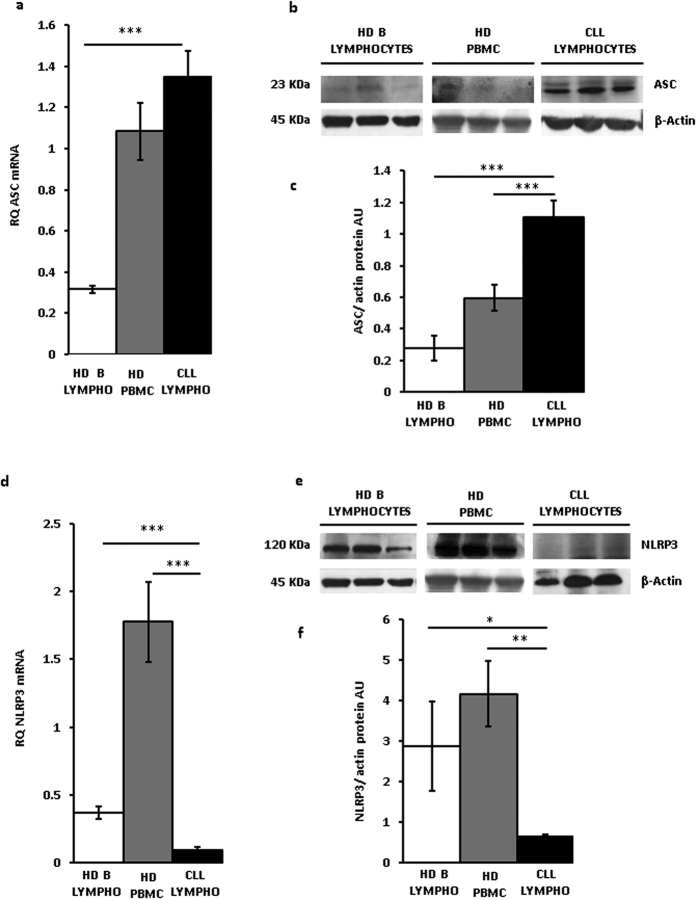
ASC and NLRP3 expression in CLL lymphocytes. Peripheral CLL lymphocytes and B lymphocytes and PBMCs from healthy donors were isolated and qRT-PCR and Western blot analysis performed as described in Methods. ASC (**a–c**) and NLRP3 (**d**–**f**) protein (**b,c,e,f**) and mRNA expression (**a**,**d**) from lymphocytes from 23 CLL patients (see Table 1), PBMCs from 12 HDs, and B lymphocytes from 5 HDs is shown. Representative Western blot (**b**,**e**)and densitometry (**c**,**f**) of CLL lymphocytes, HD PBMC and HD peripheral blood B lymphocytes from three subjects (determinations from each subject in triplicate) representative of each population are shown. Data are averages ± SEM. *p < 0.05; **p < 0.01; ***p < 0.001.

Figure 2a shows that ASC mRNA expression in CLL lymphocytes was slightly and not significantly higher than in HD PBMCs, but several fold higher relative to HD B lymphocytes. ASC protein was also overexpressed in CLL lymphocytes (Fig. 2b,c). On the contrary, and to our surprise, NLRP3 mRNA levels of CLL lymphocytes were at least 10 and 3 fold lower compared to HD PBMCs and HD B lymphocytes, respectively. NLRP3 protein levels were also significantly lower in CLL lymphocytes compared to HD lymphocytes or HD PBMC (*p < 0.05 and **<0.01 versus PBMCs and B lymphocytes, respectively, Fig. 2e,f). These data show that leukocytes from HDs, whether PBMCs or peripheral blood B lymphocytes, are characterized by low P2X7R and ASC expression and high NLRP3 levels, while CLL lymphocytes on the contrary are characterized by high P2X7R and ASC expression and low NLRP3 levels. A regression plot highlighted the inverse correlation between NLRP3 and P2X7R mRNA levels in CLL lymphocytes, although regression coefficient was low (0.2441, Figure S1). Our previous findings showed that the P2X7R promotes growth *in vitro* and *in vivo*, and in a number of experimental tumors its expression positively correlates with proliferation. Contrary to P2X7R, present data show that NLRP3 is down-modulated in leukemia cells and negatively correlates with proliferation. To properly investigate the role of NLRP3 in cell proliferation silencing experiments would be needed. However, modulation of NLRP3 expression by silencing in CLL lymphocytes is impractical as these cells cannot be easily transfected, thus we took advantage of two human leukemic cell lines, THP-1 and Ramos cells, that can be transfected and have a fast *in vitro* growth rate.

### NLRP3 modulates THP-1 cell growth

**Figure 3:**
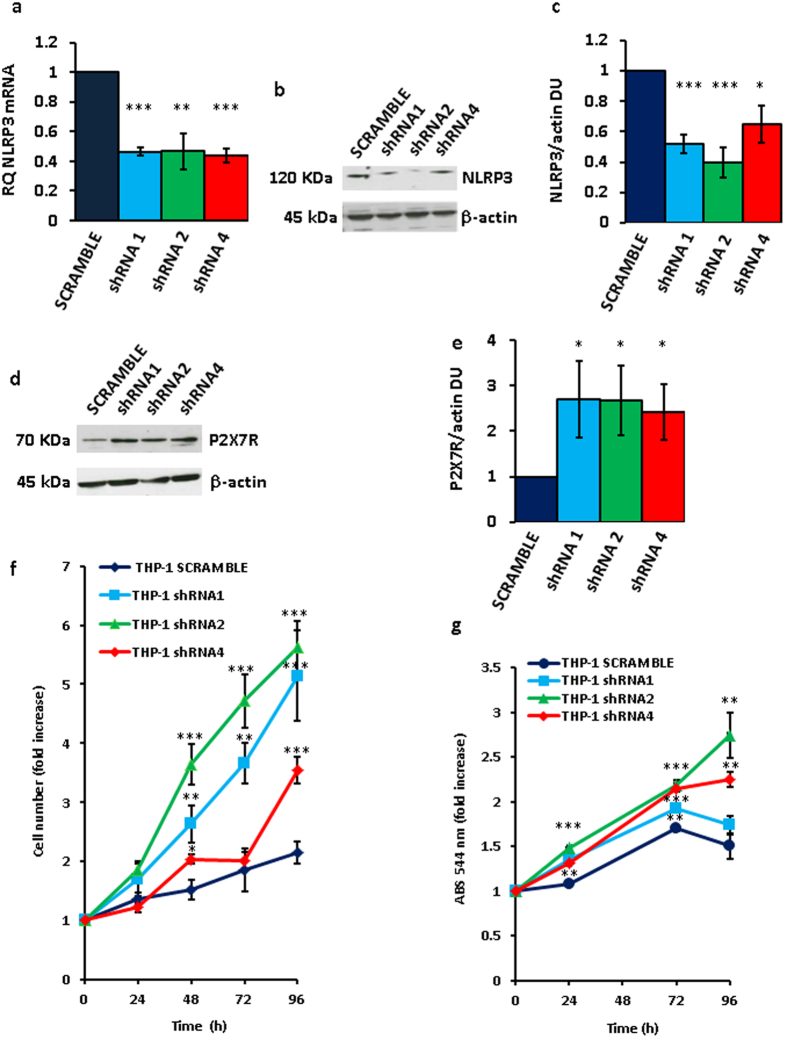
NLRP3 silencing in THP-1 cells triggers P2X7R up-regulation and stimulates growth. THP-1 cells were cultured and analyzed for NLRP3 mRNA (**a**) and protein (**b**,**c**) expression as described in Methods. NLRP3 (**a**–**c**) was silenced by HuSH-29 transfection and selected with puromycin as described in Methods. Densitometry of NLRP3 protein bands is shown in panel (**c)** (n = 3). Up-regulation of P2X7R protein in NLRP3-silenced THP-1 cells (**d**–**e**). Densitometry of P2X7R protein bands is shown in panel (**e**) (n = 3). For cell proliferation, 5 × 10^4^ cells were plated in 6-well plates in complete RPMI 1640 medium in a 5% CO_2_ incubator (37 °C) and counted (**f**) or analyzed by MTT assay (**g**) at the indicated time points. Data from triplicate determinations are shown in panels (**a,c,e**–**g**). Data are averages ± SEM. *p < 0.05; **p < 0.01; ***p < 0.001.

To down-modulate NLRP3 mRNA and protein, THP-1 cells were transfected with three different NLRP3 shRNAs (Fig. 3a–c). Very interestingly, P2X7R expression in these NLRP3- silenced cell clones was strongly enhanced (Fig. 3d,e). Our anticipation was that growth rate of NLRP3-silenced, P2X7R-overexpressing stable THP-1 clones should be faster relative to wt or mock-transfected cells. As shown in Fig. 3f,g, all NLRP3-silenced clones showed a faster growth rate relative to scramble shRNA-transfected clone over a 5 d culture assay, whether measured by counting cell number (Fig. 3f) or by the MTT assay (Fig. 3g). These observations on the one hand showed that inhibition of NLRP3 expression promoted growth, and on the other suggested that NLRP3 overexpression might negatively correlate with and even inhibit growth. To test this prediction we made several attempts to generate stable THP-1 or Ramos cell clones overexpressing NLRP3, however we were consistently unable to expand these NLRP3-overexpressing cell cultures as they systematically stopped proliferating and died few days after transfection.

**Figure 4:**
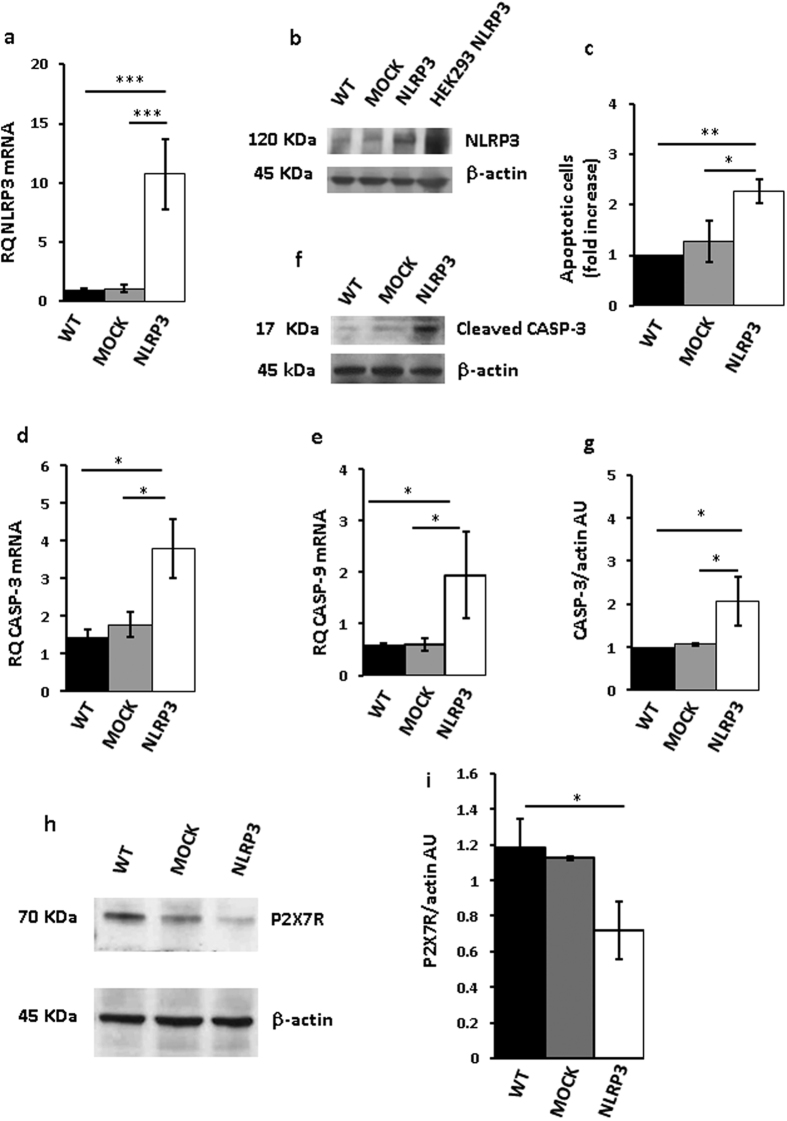
NLRP3 overexpression in THP-1 cells triggers cell death. THP-1 cells were transfected with a plasmid encoding NLRP3 as described in Methods, and then analyzed for NLRP3 mRNA and protein expression (**a**,**b**). Empty vector (mock)-transfected cells and a HEK293 clone stably transfected with NLRP3 (HEK293 NLRP3) are shown as control. Apoptotic cell number (annexin V-positive cells) is shown in panel (**c**). Caspase-3 and -9 expression is shown in panels (**d,e)**. Effect of NLRP3 transfection on caspase-3 activation (cleavage) is shown in panels (**f**,**g**). Down-modulation of P2X7R protein by NLRP3 overexpression is shown in panels (**h,i**). Data are averages ± SEM from triplicate independent determinations. *p < 0.05; **p < 0.01; ***p < 0.001.

**Figure 5:**
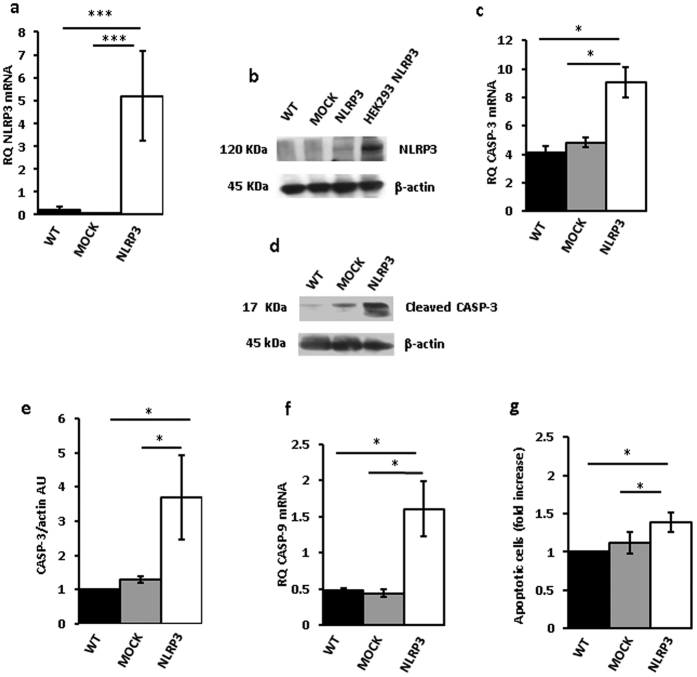
NLRP3 overexpression in Ramos cells triggers cell death. Ramos cells were transfected with a plasmid encoding NLRP3 as described in Methods, and then analyzed for NLRP3 mRNA and protein expression (**a**,**b**) Empty vector (mock)-transfected cells and a HEK293 clone stably transfected with NLRP3(HEK293NLRP3) are shown as control. Effect of NLRP3 over- expression on caspase-3 mRNA (**c**) and protein (**e**) expression and caspase-3 activation (**d**) is shown. Effect of NLRP3 transfection on caspase-9 expression (**f**) and apoptotic cell number (annexin V-positive cells) is shown in panel (**g**). Data are averages ± SEM from 3 to 4 independent determinations. *p < 0.05; ***p < 0.001.

Figure 4a,b, show that both NLRP3 protein and mRNA were strongly up-regulated in THP-1 cells 48 h after transfection. However, these cells almost at the same time underwent accelerated apoptosis, as shown by increased annexin-V staining (Fig. 4c). Expression of both caspase-9 and -3 was stimulated in NLRP3-overexpressing cells, and caspase-3 was also activated (Fig. 4d–g). Quite interestingly, P2X7R expression in these NLRP3-overexpressing cells was dramatically downmodulated (Fig. 4h,i). Transfection with the NLRP3 plasmid increased NLRP3 mRNA and protein (Fig. 5a,b), stimulated caspase-3 and -9 expression, caspase-3 activation (Fig. 5c–f), and apoptotic cell death (Fig. 5g) also in Ramos cells.

**Figure 6:**
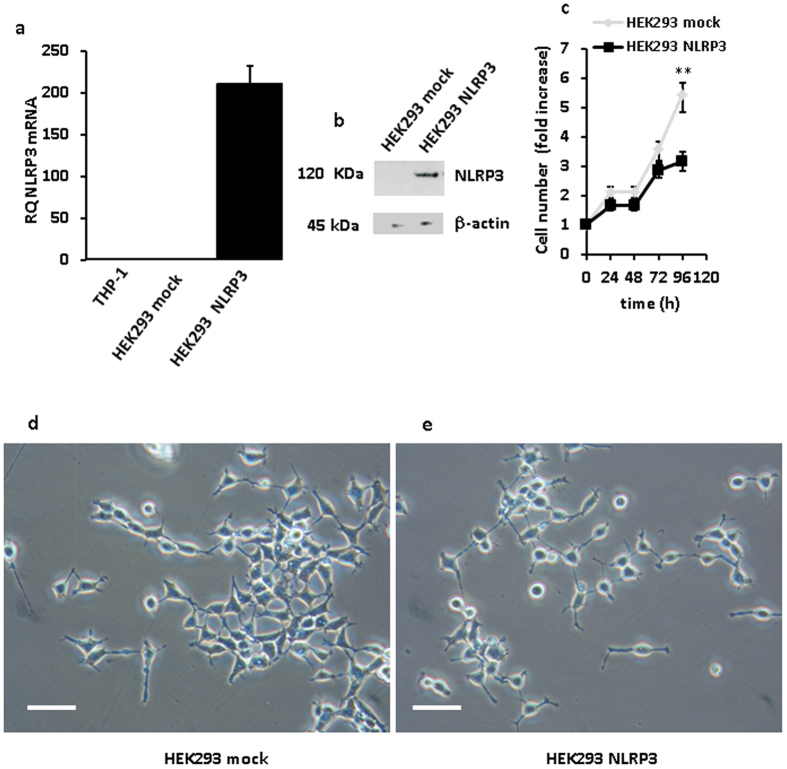
NLRP3 overexpression inhibits proliferation of HEK293 cells. HEK293 cells were transfected with a NLRP3-encoding plasmid as described in Methods, cultured in complete DMEM-F12 medium and assayed for proliferation. Panels (**a**,**b)** show level of mRNA and protein expression, respectively, in mock- and NLRP3-transfected cells. THP-1 cells (**a**) are shown as a control for baseline NLRP3 expression. Panel (**c**) shows growth kinetics of NLRP3- and mock-transfected HEK293 cells, respectively. **p < 0.01. Morphology of mock- (**d**) and NLRP3-transfected (**e**) HEK293 cells. **p < 0.01 Bar = 40 μm.

Since both THP-1 and Ramos cells proved to be extremely sensitive to the adverse effects of NLRP3 overexpression, long-term effects of NLRP3 transfection were investigated in HEK293 cells. We were able to select several stable NLRP3-expressing HEK293 clones, one shown in Fig. 6. Growth rate of these stable NLRP3-expressing clones, as shown in Fig. 6c, was significantly slower (**p < 0.01) than that of HEK293 cells transfected with the empty vector. Interestingly, not only NLRP3-transfected HEK293 cells had a slower growth rate, but also showed substantial shape changes relative to mock-transfected cells. In fact, mock-transfected cells showed the spindle-like morphology typical of wt HEK293 cells, while on the contrary HEK293 overexpressing NLRP3 were rounded with numerous dendrite-like protrusions (Fig. 6d,e).

**Figure 7:**
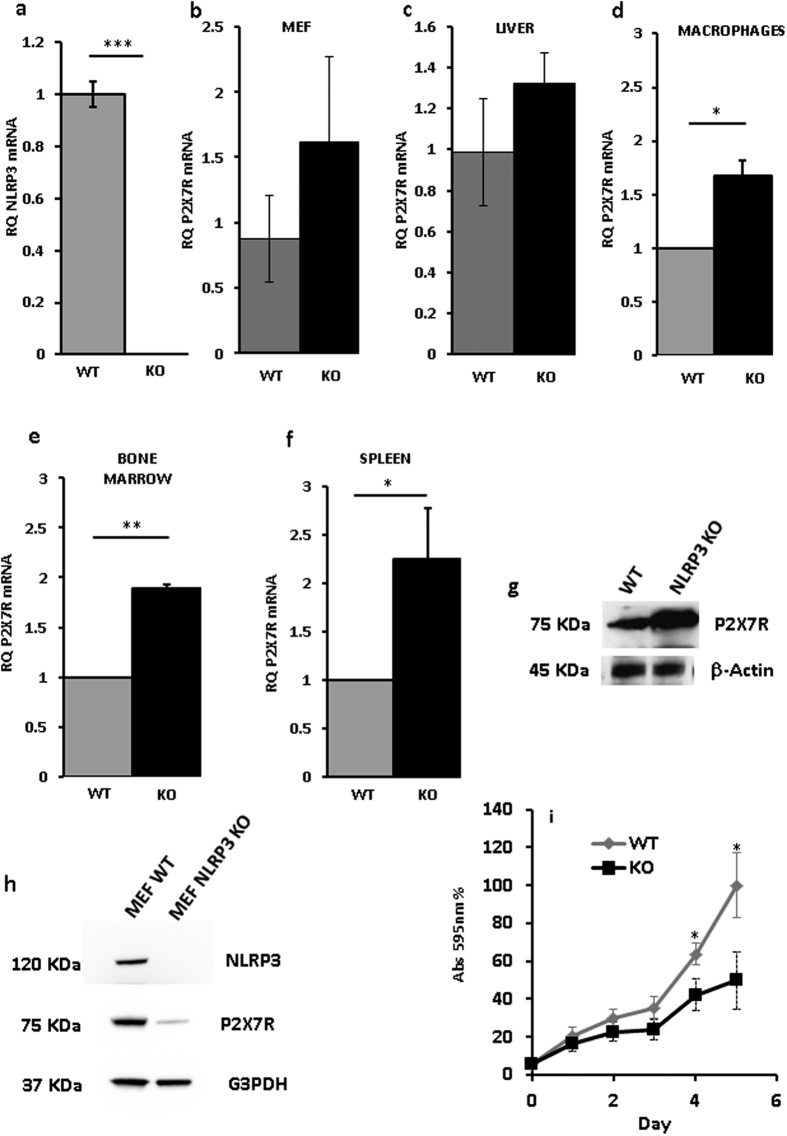
P2X7R expression in tissues and cells from *Nlrp3*^*−/−*^ mouse. NLRP3 mRNA expression in bone marrow-derived macrophages from wt and *Nlrp3*^*−/−*^ mice (**a**). P2X7R mRNA expression in MEF (**b**), liver (**c**), macrophages (**d**), bone marrow (**e**) and spleen (**f**) from wt and *Nlrp3*^*−/−*^ mice. P2X7R protein expression in spleen (**g**) and MEF (**h**) from wt and *Nlrp3*^*−/−*^ mice. Growth rate of MEFs from wt and *Nlrp3*^*−/−*^ mice (**i**). Cell were isolated and processed as described in Methods. Data are averages ± SEM from triplicate independent determinations. *p < 0.05; **p < 0.01; ***p < 0.001.

These findings strongly suggested that NLRP3 might participate in the control of cell growth and that this might involve the P2X7R. However, NLRP3 and P2X7R are mainly, although by any means not exclusively, expressed by immune cells and HEK293 are a cell line of uncertain histologic origin, thus it is possible that growth-modulatory effects of NLRP3 are restricted to lymphocytes or myelomonocytic cells. Thus, we investigated P2X7R expression in different cell populations isolated from the NLRP3-KO mouse. Figure 7a shows NLRP3 mRNA amplification from NLRP3-wt and –KO mice. Figure 7d–f, show that bone marrow-derived macrophages, bone marrow cells and spleen cells from NLRP3-KO mice expressed at least twice as much P2X7R mRNA levels compared to wt mice. Slightly higher, but not statistically significant, P2X7R levels were also present in mouse embryo fibroblasts (MEFs) and liver cells from NLRP3-deleted mice (Fig. 7b,c). However, while higher P2X7R mRNA level translated into higher P2X7R protein level in spleen lymphocytes (Fig. 7g), this was not the case in MEF cells, as in these latter cells P2X7R was strongly down-modulated (Fig. 7h). These data show that up-modulation of P2X7R in NLRP3-null cells is a feature of cells of hematopoietic origin, while P2X7R levels are much lower in NLRP3-KO than in NLRP3-WT fibroblasts. On the basis of the hypothesized growth-promoting role of the P2X7R, we anticipated that, due to P2X7R down-modulation and despite lack of NLRP3, NLRP3-KO MEFs should have a slower growth rate compared to NLRP3-WT MEFs. This anticipation was fulfilled as indeed NLRP3-KO MEFs showed a growth rate in culture which was about 30% to 50% than that of wt MEFs (Fig. 7i). These data suggest that NLRP3 effects on P2X7R expression and growth are cell type specific and likely restricted to cells of hematopoietic origin, and that anyway P2X7R is a key modulator of cell growth.

## Discussion

Inflammasomes have become a focus of hot interest in cell biology and immunology for their crucial role in several cell functions, most notably cytokine secretion and host-pathogen interaction^20^. Among the different subtypes so far described, the NLRP3 inflammasome is attracting increasing attention as it is a main target of DAMPs and the most active intracellular organelle responsible for maturation of cytokines of the IL-1β family. NLRP3 is functionally closely associated to the P2X7R, and in fact P2X7R is the most potent NLRP3-activating plasma membrane receptor^2^. P2X7R makes the NLRP3 inflammasome sensitive to extracellular ATP, one of the major DAMPs released during inflammation^21^. Furthermore, other early pro-inflammatory agents, some themselves considered DAMPs such as the cathelicidin LL37 and amyloid-β, may directly interact with and stimulate P2X7R^22^,^23^, and by consequence activate the NLRP3 inflammasome.

Besides its crucial role in IL-1β processing and release, P2X7R is also well known for its cytotoxic activity and for its counterintuitive trophic activity. We and others have extensively shown that tonic, low-level, P2X7R stimulation, far from being cytotoxic, is beneficial for cell growth and proliferation^5^,^24^,^6^. In fact, and not surprisingly, most human malignant tumors overexpress P2X7R, and targeting this receptor with selective blockers slows down *in vivo* growth of several transplanted tumors. Overexpression of P2X7R in CLL is of particular interest since a known adverse prognostic factor in this leukemia is chromosome 12 trisomy^25^. Albeit chromosome 12 is known to host several genes related to cancer^26^, a pathogenetic explanation for the adverse prognostic significance of chromosome 12 trisomy in CLL is lacking. Present and previous reports documenting an increased expression and activity of the P2X7R in CLL provide a pathophysiological explanation given the compelling evidence supporting a role of this receptor in cell proliferation and metastatic spreading. The close functional association between P2X7R and NLRP3 and the known, albeit controversial, role of IL-1β in tumor progression, initially prompted us to hypothesize that NLRP3 followed in CLL lymphocytes an expression pattern similar to P2X7R, and thus was overexpressed. However, contrary to this anticipation, our present data show that NLRP3 was strikingly down-regulated at the mRNA and protein level in CLL lymphocytes compared to HD PBMCs or peripheral blood B lymphocytes. The ASC protein on the other hand, like P2X7R, was up-regulated in cells from CLL patients.

The inverse P2X7R/NLRP3 correlation suggested that high NLRP3 levels might negatively affect cell proliferation, contrary to high P2X7R levels that support proliferation. Since primary human B lymphocytes are not amenable to transfection studies, we turned as a model for transfection to THP-1 cells, a human leukemic cell line that expresses both P2X7R and NLRP3. In agreement with our prediction, NLRP3 down-modulation caused a strong acceleration of cell proliferation paralleled by an increase in P2X7R expression. Given the known growth-promoting effect of P2X7R, it is highly likely that the proliferative advantage conferred by NLRP3 suppression was due to the enhanced P2X7R expression. Identification of P2X7R overexpression as a mechanism by which NLRP3 down-modulation supports growth provides also hints as to the metabolic pathways involved as we have previously demonstrated that P2X7R overexpression increases mitochondrial matrix Ca^2+^ and potential, intracellular ATP stores, activates NFATc1 and protects from apoptosis^5^,^9^.

We then investigated the effect of NLRP3 overexpression. NLRP3 overexpression caused P2X7R down-modulation in THP-1 cells, but it was rather difficult to prove that NLRP3 overexpression had an adverse effect on cell growth as its mere transfection proved to be cytotoxic on most THP-1 and Ramos cells, making it impossible to select stably-transfected clones, as we did for the NLRP3-silenced cells. Thus, we were forced to investigate a few and more important apoptotic markers in the 48 h following transfection. During this time, apoptosis was accelerated in both THP-1 and Ramos cells, and apoptotic caspases-3 and -9 were overexpressed and activated. After 48–72 h very few transfected cells survived, thus making additional studies impossible. However, we were able to verify the long term effects of NLRP3 transfection in HEK293 cells. This cell type turned out to be much more resistant to the adverse effects of NLRP3 transfection compared to THP-1 or Ramos cells. A few stably-transfected clones were selected and their growth kinetics could be investigated. In agreement with our prediction of the anti-proliferative role of NLRP3, NLRP3-transfected cells showed a much slower growth rate compared to empty vector- transfected or wt cells, and a strikingly different morphology.

Our findings raise the provocative issue of the possible direct participation of the NLRP3 inflammasome in the control of cell proliferation. In principle, it is not surprising that NLRP3 overexpression stimulates cell death as it is well known that inflammasome activation may trigger pyroptosis, a form of lytic cell death mediated by inflammasome activation and IL-1β release^11^. However, it is indeed surprising that NLRP3 down-modulation is growth-promoting in a cell- autonomous fashion, as shown by our results. It was very recently shown that NLRP3 is repressed in human hepatocellular carcinoma compared to healthy liver cells, and that NLRP3 expression inversely correlates with liver cancer progression^16^. This finding was interpreted by the Authors as a proof that low NLRP3 levels impaired liver cell ability to sense cytoplasmic danger signals and react accordingly, thus enhancing cell susceptibility to undergo malignant transformation. In the present study we confirm that tumor cells (CLL B lymphocytes) have low NLRP3 levels, but contrary to the interpretation put forward by Wei *et al*.^16^ we show that NLRP3 expression directly affects cell growth, in a cell autonomous fashion. However, this observation cannot be generalized to cells of non-hematopoietic origin since data from NLRP3-KO mice show that the P2X7R is up-regulated in bone marrow cells, in macrophages and in spleen cells, but not in liver cells or in fibroblasts. The mechanism by which NLRP3 modulates growth might involve the P2X7R, a receptor that is known to have a dual role as cytotoxic and a growth-promoting receptor^27^, but other mechanisms might also be involved as NLRP3 transfection also down-modulate growth in HEK293 cells, which lack endogenous P2X7R expression.

These findings help place the NLRP3 inflammasome in an entirely novel context in cancer. Current view holds that NLRP3 interferes with tumor progression in virtue of its ability to modulate cytokine release and cell death (pyroptosis), and thus control inflammation. In this context, the outcome can then be different since in certain experimental settings this activity is protective against cancer development, while in other settings NLRP3 activation and IL-1β secretion are associated to a more malignant tumor phenotype. Our data expand the role of NLRP3 in B cell leukemia as a factor that is directly controlling growth.

In conclusion, the P2X7R-NLRP3 axis appears to have an increasing importance at multiple steps involved in tumor progression, from to modulation of inflammation to control of cell proliferation and cell death. These findings might be of potential interest for the identification of novel therapies against cancer.

## Methods

### Animals

C57BL/6 wild type and C57BL/6 *Nlrp3*^*−/−*^ mice, all 4 to 8-week-old males were used for primary cultures. All procedures were performed in accordance with the Italian institutional guidelines in compliance with national and international laws and policies and were approved by the Italian Ministry of Health (n 76/2013-B).

### Cell cultures and transfections

Ramos and THP-1 cells were purchased from American Type Culture Collection (ATCC, Rockville, MD, USA), and were maintained in RPMI 1640 medium supplemented with 10% heat-inactivated fetal bovine serum, L-glutamine, 100 U/ml penicillin and 100 mg/ml streptomycin (all from Celbio, Euroclone, Milano, Italy). NLRP3-silenced THP-1cell clones were transfected by electroporation with Neon^®^ Transfection System for Electroporation (Life Technologies, Grand Island, NY, USA) with predesigned HuSH-29 shRNA targeting the following sequences: AGAGAAGGCAGACCATGTGGATCTAGCCA (shRNA1, c.n. GI321521), CAGTCTGATTCAGGAGAACGAGGTCCTCT (shRNA2,c.n. GI321522); GTACGTGAGAAGCAGATTCCAGTGCATTG (shRNA4, c.n. GI321524), or with scrambled RNA cassette (c.n. TR30013), all from OriGene (Rockville, MD, USA). Stably transfected cell lines were obtained by selection with puromycin (0.5–1 μml/L, Sigma-Aldrich, Milan, Italy). HEK293 were cultured in DMEM-F12 (Sigma-Aldrich) supplemented with 10% FBS, 100 U/ml penicillin and 100 mg/ml streptomycin. HEK293NLRP3 cells were obtained by transfection with lipofectamin 2000 (Life Technologies) as per manufacturer’ s instructions. NLRP3 plasmid was provided by OriGene (c.n. RG220952). Stably-transfected cell lines were obtained by selection with G418 sulphate (0.8 mg/ml) (Calbiochem, La Jolla, CA, USA). Single cell-derived clones, were obtained by limiting dilution. For transient expression experiments, 6 × 10^6^ cells were plated in 6- well plates and used 48 h later.

### Patients

Chronic Lymphocitic Leukemia patients were recruited at the Hematology Clinic of Ferrara University Hospital according to the guidelines issued by the local Ethical Committee and in compliance with International Regulations. Patient clinical features are shown in Table 1. All patients gave their informed consent. All experimental protocols were approved by the Ethical Committee of the Ferrara University Hospital and all experiments were performed in accordance with relevant guidelines and regulations.

### PBMC and lymphocytes isolation from CLL patients and healthy donors

Blood samples from CLL patients and healthy donors were obtained from the Hematology Clinic of Ferrara University Hospital, or from the local blood bank. Blood was diluted 1:2 with PBS (Celbio), and centrifuged on Ficoll-Hypaque (GE Healthcare Life Sciences). Cells were then rinsed twice with PBS, and resuspended in 10% FBS-supplemented RPMI 1640 medium prior to experiments.

### B lymphocyte isolation from healthy donor samples

Blood samples of healthy individuals were obtained through the blood bank of the University of Torino. PBMCs were obtained by Ficoll‐Hypaque (GE Healthcare Life Sciences, Milan, Italy) centrifugation. B lymphocytes were purified through a first step of negative selection, using anti-CD3, anti-CD16, and anti-CD14 monoclonal antibodies (produced locally) and immunomagnetic bead separation (Dynabeads Sheep anti-Mouse IgG, Life Technologies) followed by positive purification using the CD19 microbeads kit [Miltenyi Biotec S.r.l., Calderara di Reno (BO), Italy]. Flow cytometric analysis showed that these cells were more than 95% CD19^+^.

### RNA isolation and qRT PCR

Total RNA was extracted with Trizol reagent (Life Technologies), and then purified with the Pure link RNA Mini Kit (Invitrogen) as per manufacturer’s instructions. RNA content was quantitated using a Nanodrop 2000 spectrophotometer (Thermo Fisher Scientific, Milan, Italy). Reverse transcription was performed by using 1 μg of total RNA extract, using the High Capacity cDNA Reverse Transcription kit (Applied Biosystem, Carlsbad, CA, USA). qRT PCR was performed in a Step One Real Time PCR System (Applied Biosystem). Two μl of cDNA were used as a template. Amplification was performed using predesigned Taqman probes (Applied Biosystem) for P2X7R, NLRP3, ASC, casp-3, casp-9 and G3PDH as a housekeeping gene. A comparative analysis using the ΔΔCt method was used to quantitate fold increase of target cDNA relative to THP-1 wild type reference sample.

### MTT assay

Growth rate of control and NLRP3-silenced cells was measured by direct cell number count and MTT assay (Invitrogen). For MTT assay, 5 × 10^4^ cells were plated into each well of a 96-well plate. Twenty four, 48, 72 and 96 h after plating 10 μl of MTT was added to each well and incubated at 37 °C for additional 4 h. Finally, 100 μl of SDS-HCl was added to each well to solubilize the precipitate. Optical density was measured at 544 nm and data were expressed as a fold increase of THP-1 scrambled control.

### Interleukin-1β measurement

PBMCs from CLL patients or HDs (5 × 10^5^ cells/ml) were plated in a 24-well plates in complete RPMI medium, and left untreated or primed overnight with 1 μg/ml of LPS (Sigma-Aldrich). IL-1β release was measured in the supernatants of PBMCs by ELISA (R&D Systems, Minneapolis, MI, USA).

### Western blot analysis

Total cell lysates were prepared in a lysis buffer (300 μM sucrose, 1 mM K_2_HPO_4_, 5.5 mM D-glucose, 20 mM Hepes) supplemented with protease inhibitors (1 mM phenylmethylsulfonyl fluoride, 1 mM benzamidine) (all by Sigma-Aldrich). Proteins were quantified using the Bradford method. Samples were loaded into 4–12% Bolt-SDS pre cast-gel (Life Technologies), and transferred onto nitrocellulose membranes (GE Healthcare Life Sciences). Membranes were incubated overnight with the primary Abs at 4 °C. The anti-P2X7R Ab (Sigma-Aldrich) was used at a dilution of 1:200 in TBS-t (50 mM Tris, 150 mM NaCl, 0.1% Tween 20, pH 7.6) supplemented with 3% non- fat milk (Biorad, Copenhagen, Denmark) and 0.5% BSA (Sigma-Aldrich). The anti-ASC Ab (MBL, Woburn, MA,USA) was diluted 1:200 in PBS supplemented with 1% skimmed milk. The anti-NLRP3 Ab (Abcam, Cambridge, UK) was diluited 1:500 in PBS. The anti-caspase-3 Ab (Cell Signalling, Milano, Italy) was diluited 1:1000 in TBS-t supplemented with 5% non-fat milk (Biorad). The anti-actin Ab (Sigma-Aldrich) was diluited 1:1000 in TBS-t. Membranes were incubated with secondary goat anti-rabbit or anti-mouse HRP-conjugated Abs (Biorad) at a 1:3000 dilution in TBS-t buffer, or in PBS-t (PBS supplemented with 0.1% Tween 20) for 1 h at room temperature. Detection was performed with the Luminata™ Western chemiluminescent HRP kit (Merck S.p.A., Milan, Italy). Densitometric analysis of the protein bands was perfomed with ImageJ software. Band density was normalized over the actin band.

### Measurement of Apoptosis

Forty eight hours after transfection cells were centrifuged, washed with PBS (Celbio), and resuspended in 500 μl of labelling medium containing 5 μl of annexin V-Cy3 solution (Biovision, Milpitas,CA,USA). After 10 min of incubation cell fluorescence was measured with a Tali™ image-based cytometer (Invitrogen).

### Isolation and differentiation of murine bone marrow cells and bone marrow-derived macrophage

Bone marrow cells were isolated by flushing femurs and tibia of 4-to 8-week old C57Bl/6 mice with complete RPMI medium. The cell suspension was collected, centrifuged for 10 min at 150xg and re-suspended for 5 minutes at room temperature in red blood cell lysis buffer (Sigma-Aldrich). Reaction was stopped by adding RPMI, cells were centrifuged, and resuspended in Trizol for mRNA analysis. Alternatively, after incubation in red blood cell lysis buffer, bone marrow cells were re-suspended in M-CSF-supplemented (200 ng/ml) RPMI for macrophage differentiation, and seeded in Petri dishes at a concentration of 2.5 × 10^5^ cells/ml. After 7 days cells were detached using cold PBS supplemented with 2 mM EDTA and counted.

### Isolation of spleen cells

Spleens were isolated, homogenized by careful pulping and treated with red blood cell lysis buffer for 5 minutes at room temperature to remove erythrocytes. The cell suspension was then supplemented with RPMI, centrifuged for 10 minutes at 150xg, filtered through at 70 μm-cell strainer (Becton Dickinson, Franklin Lakes, NJ, USA), rinsed twice with RPMI and finally re-suspended in the same medium at a concentration of 20×10^6^ cells/ml.

### Isolation of mouse embryo fibroblasts (MEFs) and analysis of proliferation

WT and *Nlrp3*^*−/−*^ MEFs were isolated from pregnant mice at 13 or 14 day post coitum. Mice were sacrificed by cervical dislocation. Embryos were harvested, MEFs were isolated as previously described and cultured in DMEM medium supplemented with 10% FBS, penicillin/streptomycin, and 2 mM L-glutamine^28^. Growth curves were generated by seeding 10^4^ cells per well in 12-well plates. Plates were stained with crystal violet at each indicated time point. Each experiment was performed in triplicate. Crystal violet was extracted with 10% acetic acid followed by plate reading at 590 nm.

### Statistical analysis

All data are shown as means ± standard error of the mean (SEM). Test of significance was performed by Student’s t-test using GraphPad InStat software (GraphPad, San Diego, Ca, USA).

## Additional Information

**How to cite this article**: Salaro, E. *et al*. Involvement of the P2X7-NLRP3 axis in leukemic cell proliferation and death. *Sci. Rep.* **6**, 26280; doi: 10.1038/srep26280 (2016).

## Supplementary Material

Supplementary Information

## Figures and Tables

**Table 1:** Clinical features of CLL patients.

**ID PATIENT**	**SEX**	**AGE**	**CD 38**	**ZAP 70**	**IgVH**	**WBC/mm**^3^	**LYMPHOCYTES/mm**^3^	β**2-microglobulin mg/L**	**TRISOMY 12**
CLL 01	M	62	+	+	unmutated	12050	5330	5,6	+
CLL 02	F	70	+	−	unmutated	19360	14470	3,6	−
CLL 03	M	84	−	−	unmutated	74040	66640	4,04	−
CLL 04	F	58	+	+	unmutated	14160	8830	5,6	+
CLL 05	F	70	+	+	unmutated	79430	65550	4,1	−
CLL 06	M	62	−	−	mutated	98780	94400	2,04	−
CLL 07	M	61	−	+	unmutated	13470	8690	4,6	−
CLL 08	F	77	+	+	unmutated	15900	9920	6,6	+
CLL 09	F	68	+	+	unmutated	21950	18440	9	+
CLL 10	M	54	+	−	mutated	13700	7110	2,7	+
CLL 11	M	64	−	+	unmutated	14000	10220	3,1	+
CLL 12	M	75	+	+	unmutated	34310	22490	4	−
CLL 13	F	79	+	+	unmutated	14800	7460	2,3	+
CLL 14	F	74	−	−	mutated	39000	29220	2,5	−
CLL 15	F	77	+	−	unmutated	14160	8300	5,3	+
CLL 16	M	54	−	+	unmutated	12452	108330	2,2	+
CLL 17	M	72	−	−	mutated	36850	27070	6,2	−
CLL 18	M	79	−	+	unmutated	17477	16401	11	−
CLL 19	F	69	+	+	unmutated	7520	5930	2	−
CLL 20	M	70	−	+	unmutated	20810	12960	2,2	−
CLL 21	F	65	+	+	unmutated	39360	31060	2	−
CLL 22	M	46	−	+	unmutated	15770	10450	3,2	−
CLL 23	M	84	+	+	unmutated	11956	9627	5	+
