# Supplementary material for: Involvement of the P2X7-NLRP3 axis in leukemic cell proliferation and death
Source: Sci Rep. 2016 May 25;6:26280. doi: 10.1038/srep26280 (PMC4879576; doi:10.1038/srep26280)
Supplement: Supplementary Information [file srep26280-s1.pdf]

Supplementary material to: Involvement of the P2X7-NLRP3 axis in leukemic cell proliferation and death.

Authors: Erica Salaro[1], Alessia Rambaldi[1], Simonetta Falzoni[1], Francesca Saveria Amoroso[1], Alessia Franceschini[1], Alba Clara Sarti[1], Massimo Bonora[1], Francesco Cavazzini[2], Matteo Rigolin[2], Maria Ciccone[2], Valentina Audrito[3], Silvia Deaglio[3], Pablo Pelegrin[4], Paolo Pinton[1], Antonio Cuneo[2], and Francesco Di Virgilio[1*]

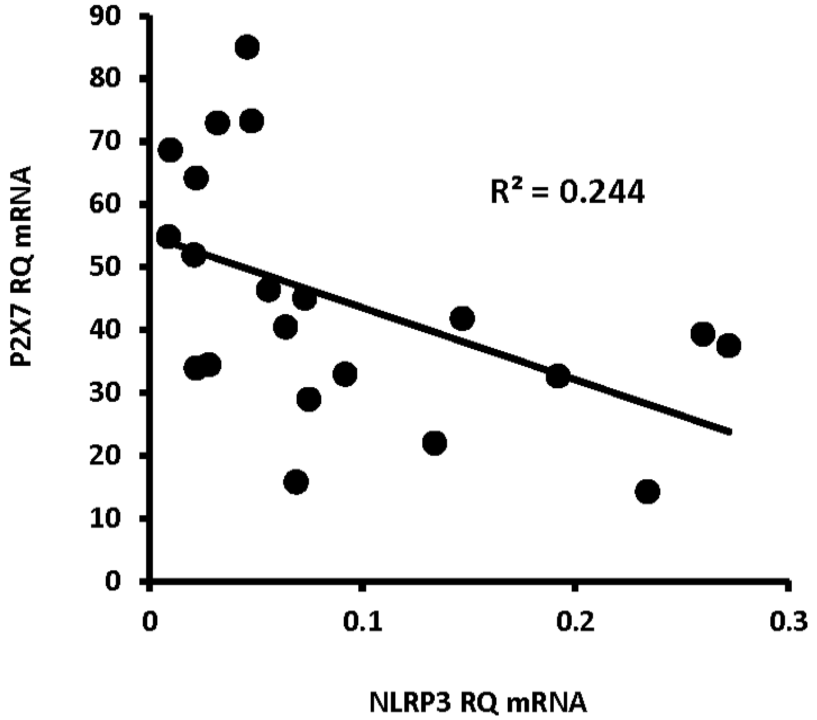
P2X7 RQ mRNA
90
80
70
60
50
40
30
20
10
0
0
0.1
0.2
0.3
NLRP3 RQ mRNA
R² = 0.244

Legend to Supplementary Figure S1

Figure S1. Correlation between P2X7 and NLRP3 mRNAs in lymphocytes from CLL patients.

Lymphocyte mRNA content was determined as described in Methods. Patients at the upper left-hand side of the graph were those with chromosome 12 trisomy, and this might explain their wide deviation from regression line. Regression coefficient (R^2) was calculated by standard regression analysis.
